# Using Simulations to Explore Sampling Distributions: An Antidote to Hasty and Extravagant Inferences

**DOI:** 10.1523/ENEURO.0339-25.2025

**Published:** 2025-10-23

**Authors:** Guillaume A. Rousselet

**Affiliations:** School of Neuroscience & Psychology, University of Glasgow, United Kingdom

**Keywords:** correlation, ERP, estimation, measurement precision, reaction times, statistical power

## Abstract

Most statistical inferences in neuroscience and psychology are based on frequentist statistics, which rely on sampling distributions: the long-run outcomes of multiple experiments, given a certain model. Yet, sampling distributions are poorly understood and rarely explicitly considered when making inferences. In this tutorial and commentary, I demonstrate how to use simulations to illustrate sampling distributions to answer simple practical questions: for instance, if we could run thousands of experiments, what would the outcome look like? What do these simulations tell us about the results from a single experiment? Such simulations can be run a priori, given expected results, or a posteriori, using existing datasets. Both approaches can help make explicit the data generating process and the sources of variability; they also reveal the large uncertainty in our experimental estimation and lead to the sobering realization that, in most situations, we should not make a big deal out of results from a single experiment. Simulations can also help demonstrate how the selection of effect sizes conditional on some arbitrary cutoff (*p* ≤ 0.05) leads to a literature filled with false positives, a powerful illustration of the damage done in part by researchers’ over-confidence in their statistical tools. The tutorial focuses on graphical descriptions and covers examples using correlation analyses, proportion data, and response latency data. All the figures and numerical values in this article can be reproduced using code available at https://github.com/GRousselet/sampdist.

## Significance Statement

We all agree that the brain is complex and that statistical modeling is hard, yet, in the literature, it is common to see data from a single experiment analyzed using a simplistic and inappropriate model, followed by sweeping claims based on statistical significance. A potent cure to mindless statistical rituals and over-confidence in our tools is to consider a long-run perspective: what could the results look like if we carried out not one, but thousands of experiments? We can answer this question by using relatively simple simulations to learn more about our data and our tools. This tutorial will help you start on this worthwhile journey.

## Introduction

The typical neuroscience or psychology article reports data from one or a few experiments. Inferences are usually made about some unspecified population using frequentist statistics. An arbitrary cutoff is then used to dichotomize effects as significant or not, and to claim a discovery, irrespective of other sources of information (McShane et al., 2019). This mostly mindless ritual gives the illusion of certainty despite the noise and variability inherent to data collection and analysis (Gigerenzer and Marewski, 2015; Gelman, 2018; Yarkoni, 2022). A large part of the so-called replication crisis is probably due to this over-confidence, stemming from the erroneous belief that statistical methods can deliver the truth. In practice, most discoveries can only be made in the long-run, following an extensive program of research (Morey, 2018). This long-run perspective is often lost in the flashy short papers that claim a discovery based on one noisy sample. It is therefore essential for researchers to be aware of this long-run perspective. One of the most efficient ways to achieve this goal is to perform simulations (Morris et al., 2019; DeBruine and Barr, 2021; DeBruine, 2025). Here we look at sampling distributions to consider results not from one experiment but from thousands of them. This can be done using synthetic or real data, and the resulting sampling distributions can be used to answer useful questions. In this article, after defining sampling distributions, I propose a series of examples exploring estimation variability across simulated experiments. We will consider analyses of correlations, proportion data, and two types of response latency data. The examples are relatively simple, but they help demonstrate the potentially large benefits of learning to and spending the time to write a few lines of code to simulate experimental results. By engaging in this type of exercise, we must explicitly consider the data generating process, including the experimental design and the shape of the population distributions we sample from when we carry out experiments (Gelman et al., 2020; McElreath, 2020). We also directly visualize the effect of sample sizes. As a result, the simulation process should ultimately lead to better planning of experiments and also a better understanding and acceptance of uncertainty, a more modest interpretation of results, and a healthy skepticism of published results (Vasishth and Gelman, 2021).

## Sampling Distributions

Sampling distributions are at the core of inferential frequentist statistics. Indeed, frequentist statistics deal with the long-run outcomes of imaginary experiments (Wagenmakers, 2007; Kruschke, 2013). For instance, when performing a *t* test, we use the sampling distribution of *t* values assuming that there is no effect to compute the probability of observing a *t* value at least as large as the one we obtained based on our sample—the so-called *p* value (Greenland et al., 2016). The point is that all users of frequentist statistics already use sampling distributions, although they might not know it (Bayesians also use them when setting informed priors from the literature). But there is more to sampling distributions than *t* tests and other standardized statistics. Sampling distributions can be used to learn about the long-run behavior of certain quantities over many experiments. For instance, we can ask what we can expect to observe if we did thousands of experiments.

**Figure 1.**
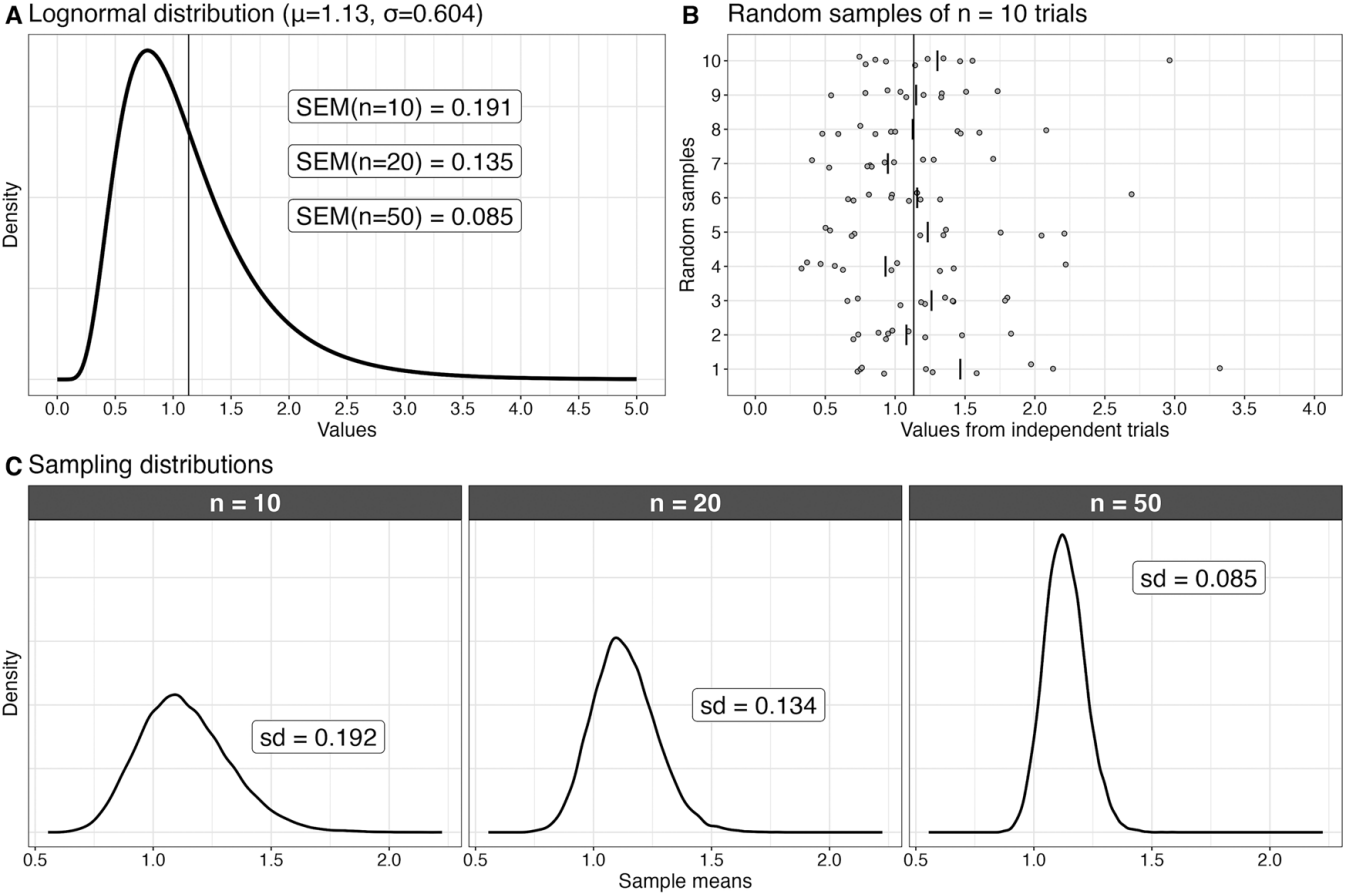
Sampling from a lognormal distribution. ***A***, Lognormal distribution with parameters log *μ* = 0, log *σ* = 0.5. The corresponding population mean and standard deviation are *μ* = 1.13, *σ* = 0.604. The vertical line marks *μ*. The insets contain the standard errors of the mean (SEM) for three sample sizes. ***B***, Scatterplots of 10 random samples from ***A***. The long vertical line marks the population mean. Each disk is an observation. The short vertical lines mark the sample means. ***C***, Sampling distributions of the mean for 20,000 samples of sizes *n* = 10, *n* = 20, *n* = 50 observations. For each sample size, the standard deviation of the sampling distribution of the sample mean is equal to the SEM. This figure was created using the R notebook *lognormal.Rmd*.

A sampling distribution is essentially the outcome of a simulation in which the same experiment is carried out many times (Baguley, 2012). To illustrate, let's imagine that we perform experiments in which we sample from the skewed distribution in Figure 1*A*. This distribution has mean *μ* = 1.13 and standard deviation *σ* = 0.604, and like many quantities we measure in neuroscience and psychology, it takes only positive values. From these population parameters, the standard error of the mean (SEM) is defined as $\sigma /\sqrt{n}$; thus 0.191 for *n* = 10, 0.135 for *n* = 20, 0.085 for *n* = 50. The *σ* population value is not usually observable, so it is estimated from random samples of observations (aka experiments), using the sample standard deviation (SD), such that $\mathrm{SEM}=\mathrm{SD}/\sqrt{n}$.

Random samples of 10 observations are illustrated in Figure 1*B*. Each of these samples has a different sample mean, a different sample SD, and therefore a different estimated SEM. This seems obvious, but panel B reminds us that there can be a lot of variability across experiments measuring the same phenomenon, something that is easy to forget when considering the outcome of a single experiment.

Instead of a few virtual experiments, we can perform many of them to see how the sample mean is distributed in the long run. Figure 1*C* shows the outcome of 20,000 experiments in which we randomly sample *n* observations from a standard lognormal population (Fig. 1*A*) and compute the sample mean. The standard deviations of these sampling distributions are almost equal to the population SEMs (compare insets in panels A and C—the small differences are due to the limited number of simulation iterations). This is because the two quantities are the same. The SEM is simply the SD of the sampling distribution of the sample mean (this video explains clearly sampling distributions and the SEM: https://www.youtube.com/watch?v=J1twbrHel3o). Hence, whereas the sample SD tells us about the variability of observations about the sample mean, SEM tells us about the variability of the sample mean about the population mean (Howell, 2013).

We can also make two important observations from the sampling distributions. First, they are skewed when drawn from a lognormal population, even for *n* = 50, which violates the assumption of the one-sample *t* test, leading to inaccurate *p* values and confidence intervals (Rousselet et al., 2023; Wilcox and Rousselet, 2023). Second, with increasing sample size, the distributions get narrower, which means that each sample mean is on average closer to the population mean. And that's the main reason to use large sample sizes: to get, on average, closer to the truth. Hence, computing sampling distributions and illustrating them can provide intuitive descriptions of the long-run behavior of a quantity, from which we can assess if our models are appropriate and help us grasp how far off our experimental results could be from the truth. In the rest of this article, we focus on the second aspect by exploring sampling distributions in a series of examples, looking at analyses of correlations, proportion data, and response latency measurements.

## Correlation Analyses

Although correlation analyses may not be the optimal way to answer the most meaningful questions about one's data (Baguley, 2009, 2010), they are omnipresent in the literature. Like the more flexible and meaningful regression analyses, correlation analyses suffer from two major issues. First, standard techniques are not generally robust to violations of their assumptions. Such violations can considerably affect the estimation of correlation and regression coefficients (Wilcox and Rousselet, 2023). Second, the sample size strongly affects the precision of the estimates, and small sample sizes may yield highly inaccurate estimates. Here we focus on the second issue as it pertains to correlation analyses.

The problem with small sample sizes in correlation analyses is not new. Approximately 16 years ago, Vul et al. warned the community about the prevalence of false positives in brain–behavior correlation analyses. They argued that standard practices led to the so-called voodoo correlations (Vul et al., 2009a,b). In one of several replies to their article, Yarkoni suggested that the main cause of voodoo correlations was the lack of power of studies with small sample sizes (Yarkoni, 2009). Yarkoni's argument is this: correlation estimates from small samples are imprecise, such that even if samples are taken from a population with a zero correlation, large correlation estimates can be expected by chance. However, in small samples, only extreme correlation estimates yield statistically significant tests. Thus, lack of power, combined with publication bias toward new, unexpected positive results, can easily lead to a literature replete with overestimates (Forstmeier et al., 2016). Although this problem is well documented, in my experience, it is still very common to see articles with sample sizes too small to estimate correlations with sufficient precision.

**Figure 2.**
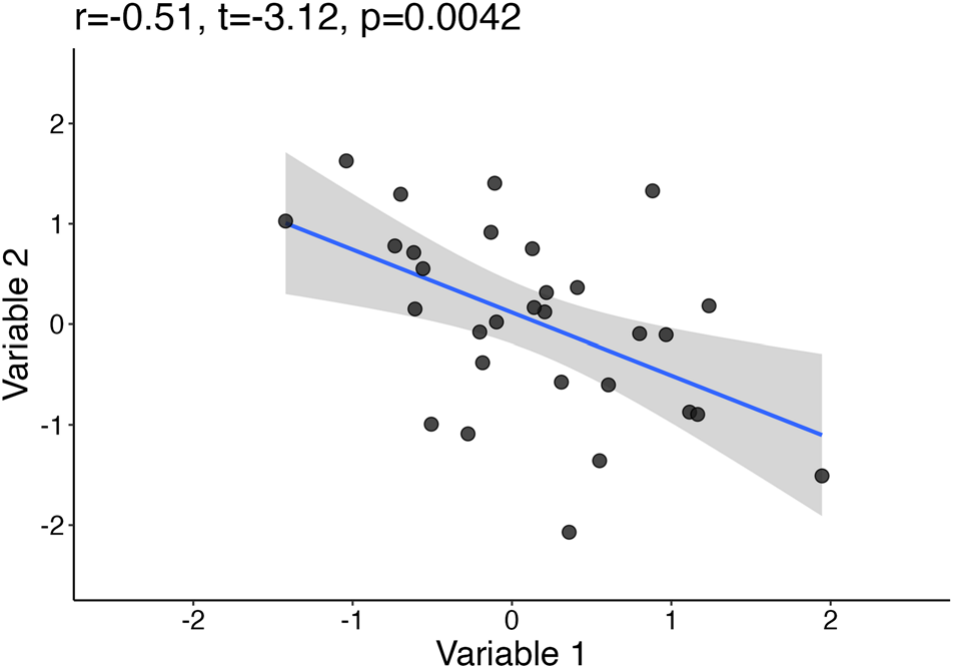
Nice looking but random correlation? The sample size is *n* = 30. The bivariate sample comes from a population with a true effect size *ρ* of 0. In other words, we are looking at sampling noise. This figure was created using the R notebook *corr_sim.Rmd*.

Let's illustrate the problems associated with small sample sizes by looking at sampling distributions. But first, let's start with the example in Figure 2. Sample size is 30, and the estimated Pearson's correlation coefficient *r* is −0.51. It seems we have discovered a relatively strong association between variables 1 and 2! Unfortunately, this effect will not replicate, because the bivariate data in the scatterplot were sampled from a population with zero correlation (*ρ* = 0, the Greek letter *ρ* represents the population correlation coefficient). So the true effect is zero, but our sample leads us to believe otherwise. There is nothing new here: inaccurate effect sizes are a natural outcome of studies with small sample sizes. The problem only gets worse once we add questionable research practices (such as selective reporting) and incentives to publish novel, positive results to the equation. (To be fair, the example in Fig. 2 is the outcome of selective reporting: I generated all the correlations among 20 samples and picked the samples associated with the highest correlation to make a point!)

**Figure 3.**
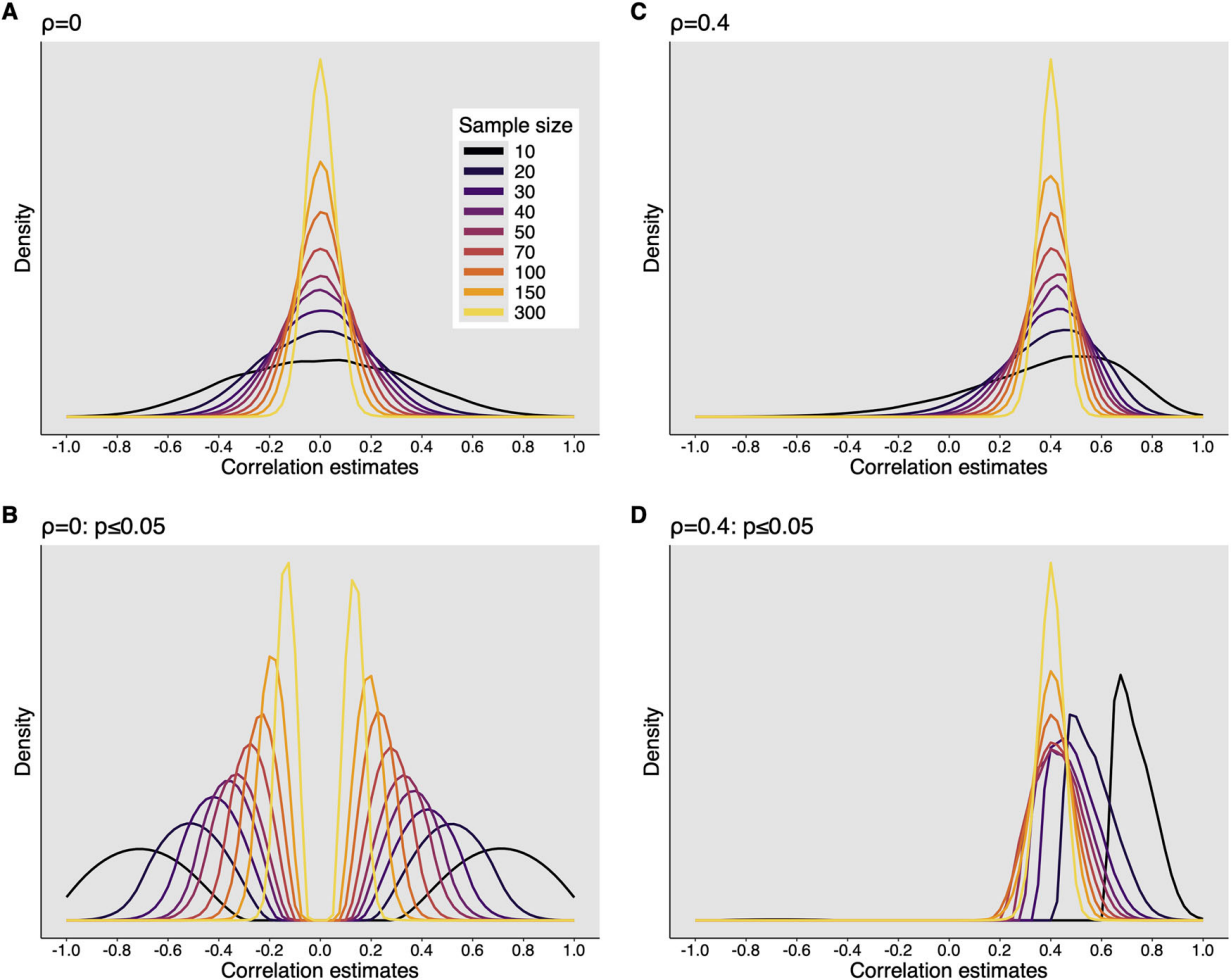
Examples of sampling distributions of correlation coefficients. Results are presented for Pearson's correlation. Similar results would be obtained using Spearman's correlation or some other measure of association. ***A***, Sampling distribution for *ρ* = 0. ***B***, Sampling distribution for *ρ* = 0, given *p* ≤ 0.05. ***C***, Sampling distribution for *ρ* = 0.4. ***D***, Sampling distribution for *ρ* = 0.4, given *p* ≤ 0.05. This figure was created using the R notebook *corr_sim.Rmd*.

The effect size inflation with small sample sizes might seem counter-intuitive because, if a study lacks power, surely the true effect must be very strong to show up with such a small sample size. However appealing, this conclusion is wrong because it ignores a critical aspect of studies with small sample sizes: they are associated with large sampling variability, which means that estimation precision is poor. This error in reasoning has been described as the “which does not kill statistical significance makes it stronger” fallacy (Loken and Gelman, 2017). The problem becomes clear when we draw samples of different sizes from a normal bivariate population with a known population Pearson’s correlation *ρ* of 0. The sampling distributions of the estimates of *ρ* for different sample sizes are shown in Figure 3*A*, which illustrates that the sample estimates often differ considerably from the population value, particularly in small samples. And the situation gets worse when we compare correlation coefficients (Rousselet et al., 2023).

Sampling distributions tell us about the behavior of a statistic in the long run, if we did many studies. With larger sample sizes, the sampling distributions are narrower, which means that the individual estimates tend to be more precise. However, a typical article reports only one correlation estimate, which could be completely off. So what sample size should we use to get a precise estimate? The answer depends on the following:

1. The shape of the univariate and bivariate distributions
2. The quantity we want to estimate (by default Pearson, a poor choice)
3. The true effect size (the larger the effect, the fewer trials are needed—see below)
4. The precision we want to afford

**Figure 4.**
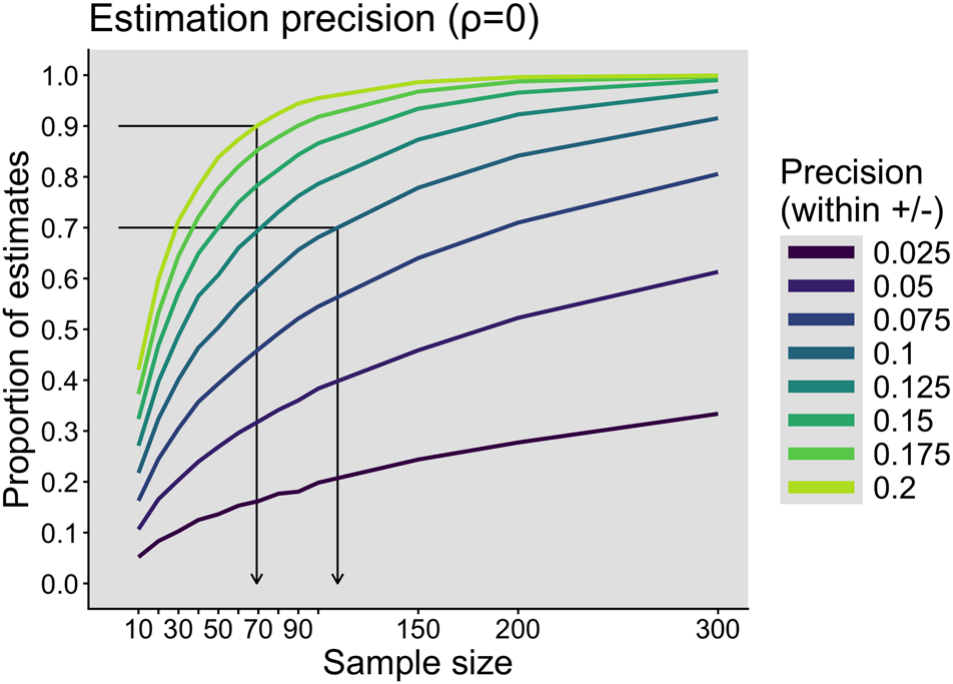
Correlation: estimation precision. Proportions of estimates near the true value (*ρ* = 0), for different sample sizes, and for different levels of precision. This figure was created using the R notebook *corr_sim.Rmd*. Readers interested in matching curves for statistical power will find them in the R notebook *corr_power.Rmd*.

For the sampling distributions in Figure 3*A*, we can calculate the proportion of correlation estimates that are within a certain margin from the population correlation (*ρ* = 0). For instance, as shown by the black arrows in Figure 4:

1. For 70% of estimates to be within 0.1 of the true correlation value (between *r* = −0.1 and 0.1), we need at least 110 observations.
2. For 90% of estimates to be within 0.2 of the true correlation value (between *r* = −0.2 and 0.2), we need at least 69 observations.

Using the full curves in Figure 4, researchers can consider different thresholds and reflect on the trade-off between sample size and measurement precision. The bottom line is that even if we're willing to settle for imprecise estimates (up to 0.2 from a true value of 0), we need plenty of observations to achieve this precision often enough—again, there is no guarantee whatsoever for any particular experiment we conduct. This approach fits well with a growing literature that advocates planning experiments for estimation precision rather than the traditional goal of power (Maxwell et al., 2008; Bland, 2009; Schönbrodt and Perugini, 2013; Gelman and Carlin, 2014; Peters and Crutzen, 2017; Trafimow and MacDonald, 2017; Rothman and Greenland, 2018; Trafimow et al., 2019). Researchers interested in estimation accuracy are also encouraged to consider robust alternatives to Pearson's correlation (Pernet et al., 2013; Wilcox and Rousselet, 2023).

When planning experiments, it is important to realize that if we meticulously survey the literature, we are very unlikely to obtain curves similar to the ones plotted in Figure 3*A*,*C*, because the literature almost certainly provides a biased estimation of the true population correlations (Ioannidis, 2008; Gelman and Weakliem, 2009). Indeed, consider what happens to a literature in which it is exceedingly difficult to publish nonsignificant findings (Smaldino and McElreath, 2016). We can illustrate this problem by looking at the sampling distribution of only those correlation coefficients associated with statistical significance (Fig. 3*B*).

For a correlation estimate to be significant, its absolute value needs to be large—more so for small samples. Selecting for a significant correlation coefficient therefore removes the part of the sampling distribution around *r* = 0 and only retains the more extreme values. If authors, reviewers, and editors are biased against nonsignificant findings, this means that readers only get to see an upwardly biased portion of the total distribution of correlation estimates. This, in turn, means that if researchers base their power analyses on the effect sizes encountered in the literature, they are likely to overestimate their targeted effect size and hence the power of their program of research.

**Figure 5.**
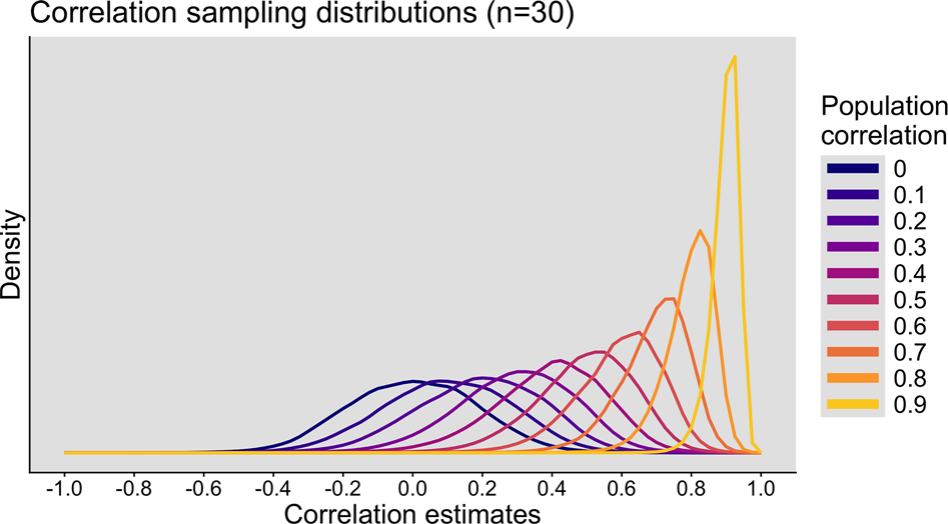
Correlation sampling distributions as a function of the population correlation. The sample size is always *n* = 30. This figure was created using the R notebook *corr_sim.Rmd*.

So far, we have considered samples from a population with zero correlation, such that any large positive or negative correlations were due to random sampling. Let us see what happens when there is a nonzero effect for a fixed sample size of 30. As Figure 5 shows, the modes of the sampling distributions increase with increasing population correlations, whereas their spreads decrease.

Consider in more detail the sampling distributions for *ρ* = 0.4 (Fig. 3*C*). The sampling distributions for *n* < 50 are negatively skewed. Consequently, the typical experiment will tend to overestimate the true value (distribution mode shifted to the right). If we report only correlations associated with *p* ≤ 0.05, the distributions look very different (Fig. 3*D*). Again, with small sample sizes, the estimates are inflated, albeit in the correct direction. There is nevertheless a small number of large negative correlations: indeed, in 0.77% of simulations, even though the population value was 0.4, a large and *p* ≤ 0.05 negative correlation was obtained—also known as a directional or type III error (Shaffer, 2002).

The main lesson from this section is that the minimum sample size needed to estimate a particular association is probably much larger than we tend to think. And this is not just a point about statistics, because the size of the true correlations we can expect in real data is also probably much weaker than often reported based on small sample sizes. For instance, large *n* studies and meta-analyses in the social sciences suggest that correlations between 0.2 and 0.4 should be considered relatively large, and weaker correlations are much more common (Schönbrodt and Perugini, 2013; Plonsky and Oswald, 2014; Frey et al., 2017), with some replication attempts demonstrating much smaller effect sizes when using larger samples than in the original studies (Zhang et al., 2018; Cai et al., 2019). Also, because the literature is biased toward positive results, meta-analyses tend to over-estimate the population effect sizes. In brain imaging, a study estimated correlations between resting-state fMRI data and personality traits across 884 participants (Dubois et al., 2018). The largest *r* was 0.27. And some of the results were highly susceptible to preprocessing methods, an analytical flexibility that could be exploited to find and only report the best possible outcome, contributing to inflated estimates in the literature. In another study involving 5,216 subjects, correlations between several brain structural measurements and two cognitive tasks were all inferior to 0.20 (Ritchie et al., 2018). A meta-analysis of 88 studies (8,036 subjects) of correlations between brain volume and IQ suggests an overall effect of *r* = 0.24 (Pietschnig et al., 2015). And a preregistered study using a larger sample size suggests that even lower correlations are more realistic (Nave et al., 2019). Thus, in the absence of large-sample replications, large correlations are best taken with a grain of salt. There are, however, a few notable exceptions where large effect sizes are expected, such as test–retest assessment and the comparison of related tests or measurements.

## Proportion Data

By looking at sampling distributions of correlation coefficients, we learnt important lessons about the relationship between estimation precision and sample size and how conditioning on arbitrary cutoffs can dramatically bias effect sizes in the literature. We now turn our attention to another type of popular analyses, that of proportion data. Such data are common in both animal and human experiments that involve behavioral measurements to assess performance (proportion of correct trials in memory and navigation tasks for instance) or when a measurement is expressed as a proportion of another one (experimental group relative to a control group for instance). This section will again help us illustrate the importance of sample sizes but also highlight another important aspect of simulations: to be explicit about the data generating process.

**Figure 6.**
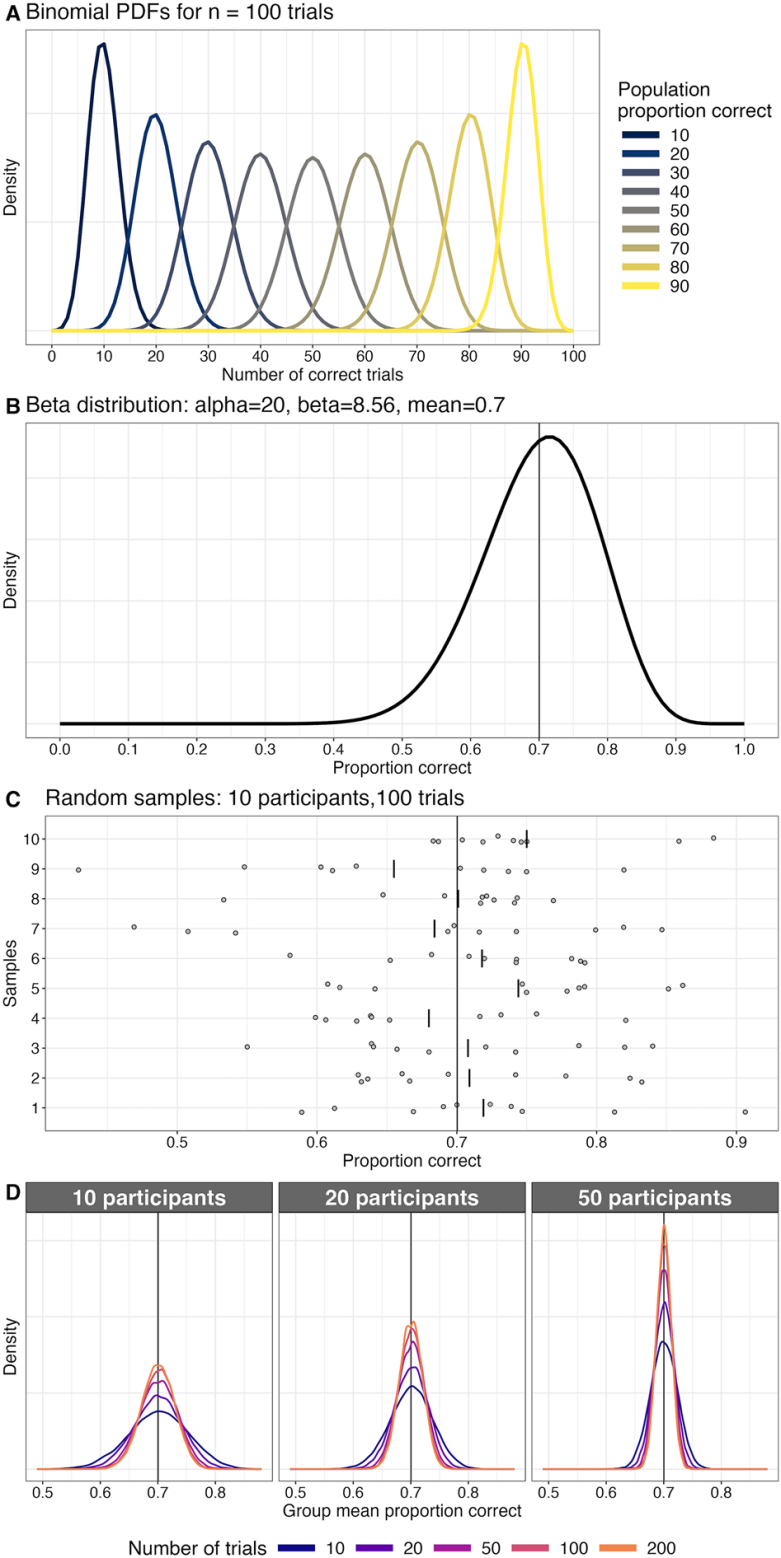
Percent correct data. ***A***, Binomial sampling distributions. PDFs, probability density functions. ***B***, Beta sampling distribution of proportion correct, with mean 0.7 (vertical line). ***C***, Proportion correct results from 10 random samples from 10 participants. For each participant, the mean percent correct was sampled from the beta distribution in panel ***B***. Then 100 trials were sampled from the corresponding binomial distribution. The long vertical line marks the population value. For each sample, each disk corresponds to the average of 100 trials for a participant and the short vertical line indicates the sample mean across participants. ***D***, Sampling distributions for group results for different numbers of trials (color coded) and different numbers of participants. For each combination of parameters, the distributions are based on a simulation with 20,000 iterations. The vertical line marks the population value. This figure was created using the R notebook *pc.Rmd*.

Let's consider the simulated data in Figure 6. Panel A illustrates the theoretical relative probabilities of observing different proportions of correct responses for different true performance levels. For instance, imagine a participant who has a true performance level of 10% correct. Also imagine that we ask the participant to perform 100 trials. What will be the participant's actual performance across all these trials? More importantly, what range of actual performance can we expect across experiments? To answer this question, we simulate many experiments. The results for our 10% example are illustrated in the left-most curve in Figure 6*A*: it shows that for any given experiment, the number of correct trials ranges from roughly 0 to 20. If instead our imaginary participant is on average 50% correct, the values across experiments range roughly from 35 to 65. Thus, the population mean percent correct and standard deviation are dependent: there is stronger variability at 50% correct and the variability decreases as the mean tends toward zero or 1.

Although this type of data are best analyzed using hierarchical (mixed-effect) models (Jaeger, 2008; Kruschke, 2014), in neuroscience and psychology correct/incorrect results and other proportion data tend to be averaged across trials for each condition and participant and entered into an ANOVA. What do the sampling distributions across participants look like? Because participants cannot be less than 0% correct or more than 100% correct, the distributions must be bounded, which rules out any continuous distribution such as the normal distribution as a good model—standard analyses such as ANOVA and *t* test assume that participants can be more than 100% correct. Instead, a good candidate to capture the shape of the data is a beta distribution, which is bounded between 0 and 1 (Kruschke, 2014; Heiss, 2021). Figure 6*B* shows an example of a beta sampling distribution with a mean of 70% correct. The distribution is negatively skewed and quite broad. Using this beta distribution, we can simulate the sampling variability expected in an experiment: each sample from the distribution corresponds to the proportion correct of a participant; then for that value, we generate trials from a binomial distribution. Figure 6*C* illustrates 10 random samples from 10 participants. For each participant, a random value from the beta distribution determined their proportion of correct trials and 100 trials were generated by sampling from the corresponding binomial distribution. Again, these random samples help us grasp the large variability inherent to experimental sampling.

Using the same approach, we can perform 20,000 simulated experiments in which we vary the number of participants and the number of trials per participant. The results are presented in Figure 6*D*. As illustrated in our correlation examples, with increasing sample sizes, precision increases (sampling distributions get narrower). The results also suggest something very useful to plan experiments: the precision increases faster with the number of participants than with the number of trials (compare panel 10 to panel 50). For instance, with 10 participants and 100 trials per participant, the probability to observe a group mean more than 5% points from the population mean is about 10%. With only 50 trials per participant, this probability is now 14%. But if we test 50 participants with 10 trials each, the probability is now only 3.5%, even though both situations involve a total of 500 observations. The increased benefit of participants over trials seem to apply to many situations (Rouder and Haaf, 2018).

## Response Latencies

### ERP onsets

Besides proportion data, another popular type of data are measures of response latencies. Here we consider two examples, one using event-related potential (ERP) data and one using manual reaction time data. As such latency data are bounded and skewed, it is important to consider sampling distributions.

**Figure 7.**
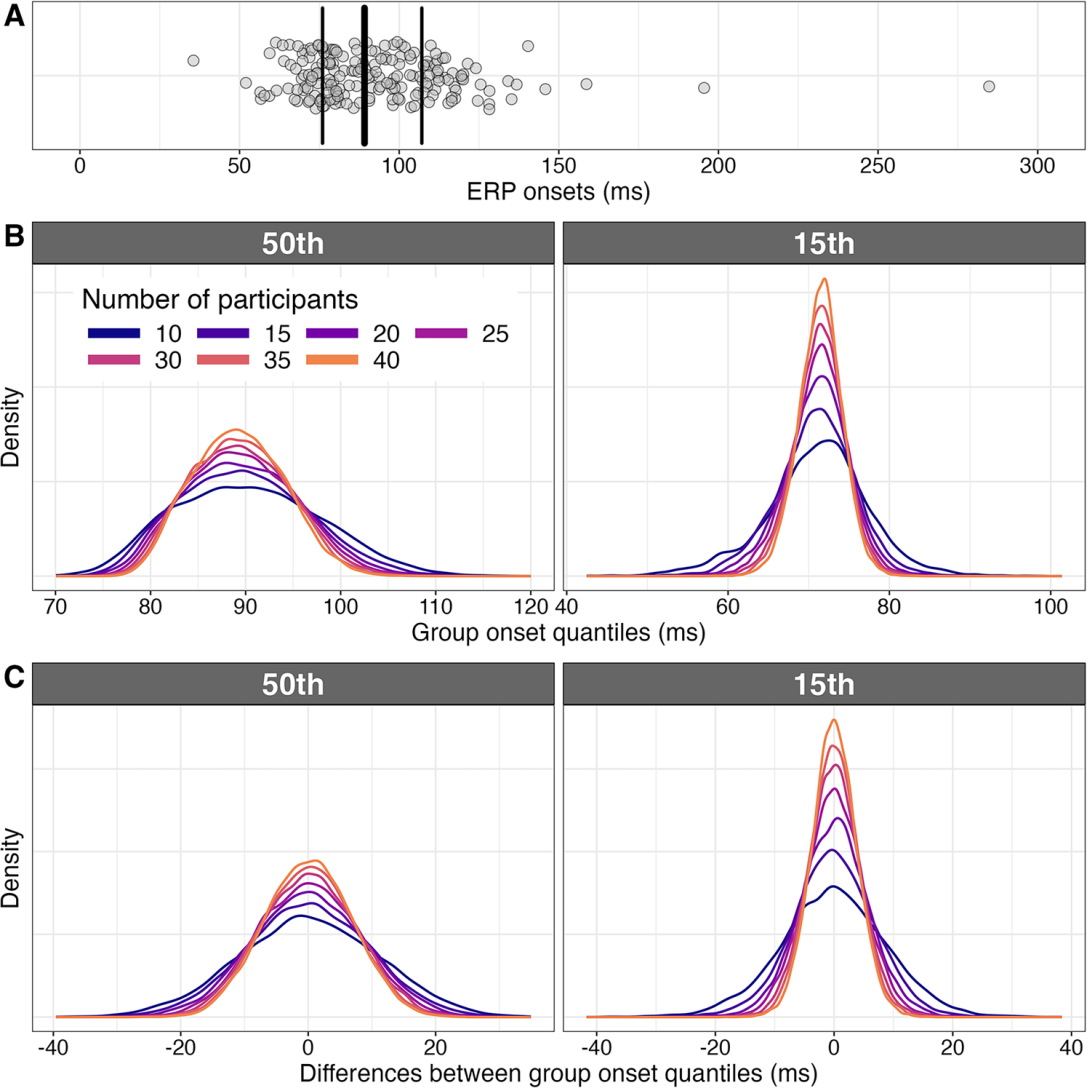
ERP onsets. ***A***, Scatterplot of 194 ERP onsets. The vertical lines mark the quartiles. ***B***, Sampling distributions of the 50th (left) and the 15th (right) onset quantiles. The quantiles were computed using the Harrell–Davis estimator, which deals better with tied values than standard estimators (Harrell and Davis, 1982). The results are based on 10,000 simulated experiments. ***C***, Sampling distributions of quantile differences between two experiments. This figure was created using the R notebook *onsets.Rmd*.

In our first example, ERP onsets (earliest differences between conditions) were estimated in 120 participants engaged in a face versus texture discrimination task; 74 of them were tested in a second session to assess test–retest reliability (Bieniek et al., 2016). Here, for convenience, we merge the two sessions to form a distribution of 194 ERP onsets, which is positively skewed (Fig. 7*A*). Because the typical ERP experiment has much fewer participants, we can use data-driven simulations to determine sampling distributions given smaller sample sizes. If we are interested in the central tendency of a skewed distribution, it can be informative to estimate the 50th quantile of the distribution—the value that splits the distribution of sorted observations in two equal parts (Rousselet and Wilcox, 2020). To estimate the sampling distributions of the 50th quantile, given our data, we sample from the distribution of onsets 10,000 times, using sample sizes from 10 to 40, incremented in steps of 5. The resulting distributions are presented in Figure 7*B*.

From these distributions, we can ask useful questions by treating the full sample as a population. For instance, what is the proportion of onsets estimates that are within ± a certain value from the population onset quantile?

Here we can motivate our choice of threshold value using the neuroscience literature. For instance, it has been estimated that on average, for short latency neurones, the transfer time between cortical areas is ∼10 ms (Nowak and Bullier, 1997). So when looking at fast visual responses, 10 ms seems like a meaningful level of precision we should care about. With a sample size of 10 participants, ∼80% of 50th quantile estimates (simulated experiments) are within ±10 ms of the population value. With a sample size of 35 participants, ∼97% of 50th quantile estimates are within ±10 ms of the population value. We can also determine the number of observations needed to achieve a certain level of estimation precision. For instance, to be within 10 ms of the full sample 50th quantile value in at least 90% of experiments, how many participants do we need? The answer is *n* = 19 participants.

There is no need to restrict our investigation to one quantile only. If the focus is on processing speed, it is of particular interest to quantify the fastest responses (Bieniek et al., 2016; Rousselet, 2025). This can be done, for instance, by estimating a lower quantile, say the 15th quantile (Fig. 7*B*).

Given the controversies about the latencies of the first face responses in the brain, it can be useful to perform a simulation in which we quantify, given our data, how far apart estimates can be between two experiments (Fig. 7*C*). We can determine, given a certain level of precision, the probability to observe similar effects in two experiments. For instance, with a sample size of 10 participants, only ∼63% of 50th quantile estimates are within ±10 ms of each other. With a sample size of 35 participants, ∼86% of 50th quantile estimates are within ±10 ms of each other. Conversely, we can determine the number of observations needed to achieve a certain performance. For instance, for 90% of pairs of experiments to generate results at most 15 ms apart, we need at least 18 observations. Such calculations might suggest that certain discrepancies in the literature are simply due to random sampling fluctuations (Wilcox and Rousselet, 2024) or might point to real differences due to other factors, such as the task or the age of the participants. Whatever the reason, simulations of sampling distributions help put results in perspective.

The sampling distributions in Figure 7*C* could also be used to calculate prediction intervals (Spence and Stanley, 2024). For instance, in the long run, over an infinite number of experiments, 95% prediction intervals calculated in one experiment will contain the estimate from a replication experiment 95% of the time. But keep in mind that prediction intervals, like confidence intervals, usually make strong parametric assumptions, such that their probability coverage is inaccurate when applied to real data (Wilcox and Rousselet, 2024).

### Reaction time data

**Figure 8.**
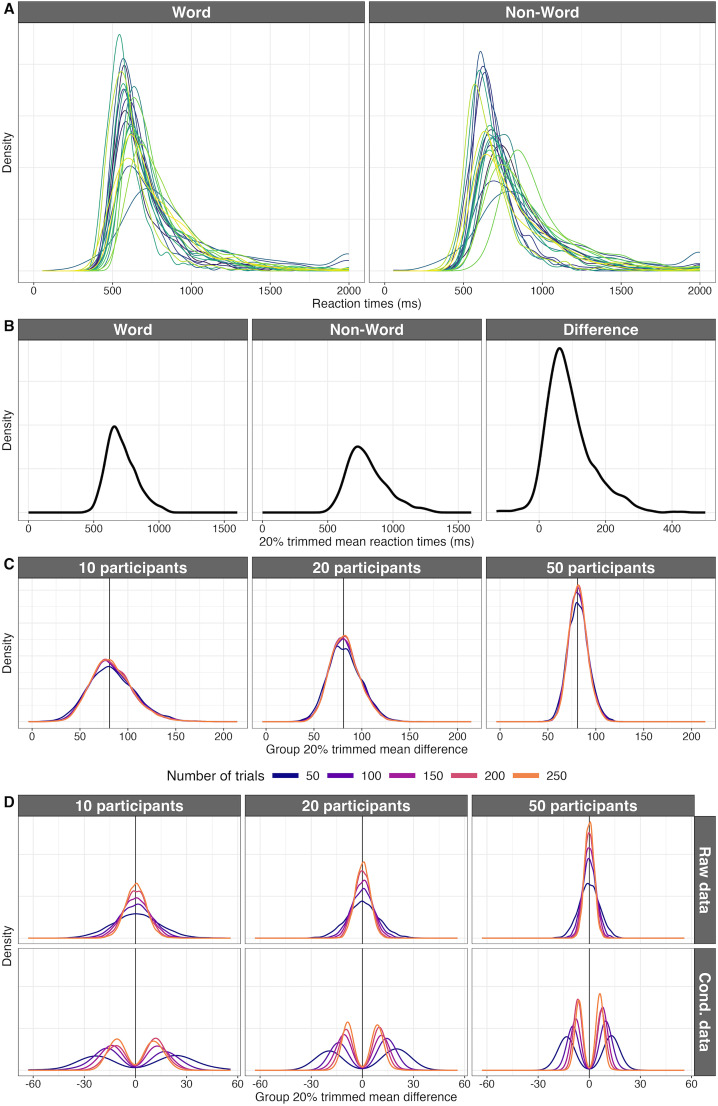
Reaction time data from the FLP dataset. ***A***, Examples of individual reaction time distributions from 20 participants, in the two conditions. ***B***, Distributions of 20% trimmed means in 959 participants: for each participant, a 20% trimmed mean was computed across trials to summarize the distribution in each condition. The right panel shows the distribution of differences between 20% trimmed means in the two conditions (Non-Word minus Word). Note how most of the differences are positive, but with very large variability. ***C***, Sampling distributions for the group 20% trimmed means, based on simulations with 5,000 iterations. The vertical line in each panel marks the population 20% trimmed mean. ***D***, Sampling distributions when there is no effect. This was simulated by pooling the two conditions together. The first row illustrates the sampling distributions as they are (raw data); the second row illustrates the same results after conditioning on *p* ≤ 0.05. This figure was created using the R notebook *flp.Rmd*.

The previous example considered a situation with only one level of analysis: for simplicity we did not consider within-participant variability because of the large amount of data involved in computing ERP onsets in each participant (Rousselet, 2025). Here we use a dataset containing a large number of participants and a large number of trials for each participant. The data are from the French Lexicon Project (Ferrand et al., 2010): manual reaction times where measured in response to words and nonwords. After discarding participants who clearly did not pay attention, we are left with 959 participants, each with ∼1,000 trials per condition (no further data cleaning was performed, such as removing outlier trials). Examples of individual reaction time distributions are shown in Figure 8*A*. The distributions are positively skewed, as expected for RT data, and participants tend to be slower in the Non-Word condition compared with the Word condition. Usually, a single number is used to summarize each individual RT distribution—a better alternative would be to use a hierarchical model (Lindeløv, 2019; Rouder and Province, 2019). From 1,000 values to 1, that's some serious data compression! In psychology, the mean is often used, but when there is skewness it can be a misleading measure of location (Trafimow et al., 2018; Rousselet and Wilcox, 2020). Instead here we use the 20% trimmed mean, which gives a better indication of the location of the typical observation and protects against the influence of outliers (Wilcox and Rousselet, 2023). To compute a 20% trimmed mean, we sort the data, remove the 20% lowest values and the 20% highest values, and average the remaining values. In that context, the median is a 50% trimmed mean and the mean is a 0% trimmed mean. We could trim more or less, but 20% works well in many situations, and this particular choice is irrelevant to the points below.

The distributions of participants’ 20% trimmed means are positively skewed in both conditions (Fig. 8*B*). And because these distributions differ in skewness, the distribution of differences between the 20% trimmed means in the two conditions is also skewed (Non-Word minus Word). The distribution of differences overlaps very little with zero: 96.2% of participants have a positive difference, meaning that they tended to be faster in the Word than the Non-Word condition.

With this large dataset, as in previous examples, we can pretend that the full dataset is our population that we're trying to estimate. We perform data-driven simulations to get sampling distributions. In this example, the sampling is hierarchical: we sample with replacement participants, and for each randomly sampled participant, we sample trials with replacement. Because there is skewness at both levels of analysis, we compute 20% trimmed means across trials for each condition and then compute 20% trimmed means across participants. This was done for simulations with 5,000 iterations in which we varied independently the number of participants and the number of trials (Fig. 8*C*).

As we saw in the previous examples, by increasing the number of participants, we gain in precision: the distributions get narrower. Interestingly, the number of trials has little effect on the width of the sampling distributions. That's because the effects are large and positive in most participants but vary a lot in magnitude across participants—see the right panel in Figure 8*B*. Thus, in this situation, it is clearly more beneficial to recruit more participants than to increase the number of trials (Rouder and Haaf, 2018).

Similarly to what we did in the previous examples, we could compute the number of trials and participants needed to achieve a certain level of estimation precision. But here we use the data to address a different question: what differences could we observe with this type of data and task if there were no difference between conditions? This situation can be simulated by pooling trials across conditions for each participant and sampling with replacement from that one pool of trials. We proceed by sampling participants with replacement, then for each randomly selected participant, we sample with replacement two sets of random response times from the response times in the two conditions mixed together.

As expected, the sampling distributions are now centered near zero, as on average, in the long run, we expect zero difference—assuming we're trying to estimate an unbiased quantity (Fig. 8*D*). But the variability across simulated experiments is large, suggesting that if results were selected based on some arbitrary cutoff, researchers could easily fool themselves into reporting false positives of large magnitude. To see what the distributions of results conditional on *p* ≤ 0.05 would look like, for each simulation, we performed a group *t* test on 20% trimmed means, a technique that requires some adjustments to the standard *t* test equation (Tukey and McLaughlin, 1963; Wilcox, 2022). The conditional distributions look very different to the raw ones (Fig. 8*D*, second row): the distributions are now bimodal with a gap around zero. Based on these distributions, what would the typical result look like? With 10 participants and 50 trials per condition, the median of the absolute group differences is ∼24 ms. Among the simulated experiments that produced results with *p* ≤ 0.05, 10% have differences at least as large as 37 ms. With 50 participants and 200 trials per condition, the median of the absolute group differences is ∼7 ms, and among the experiments with *p* ≤ 0.05, 10% produce differences at least as large as 9 ms. So, large sample sizes can strongly damp down the effect sizes of false positives reported in the literature.

Finally, it is important to consider that the type of data-driven simulations reported here is affected by the relative size difference between the population and the sample. As sample sizes get closer to the population size, sampling distributions are distorted, which biases power estimation, and the sign of the bias depends on the effect size in the population. The problem is explained in detail in Burns et al. (2025).

## Summary and Conclusions

In this article, we saw how simulations can be used to bring perspective to results from single experiments. Using simulations, we can explore potential results not from one experiment, but from thousands of them (sampling distributions). The main outcome of this process is the startling realization that in many situations, our measurements are so noisy that we should refrain from drawing strong conclusions about them. Instead, we can learn to plan more ambitious experiments from the results of simulations and develop a healthy skepticism about published results and our own.

Thus, the approach described here contributes to tackling one critical problem faced by many researchers: the over-confidence fueled by the irrational belief that statistical tests can deliver certainty in the face of measurement noise and sampling variability. More than a crisis of replicability, I would argue that we have a crisis of over-confidence, one that can be tackled not by teaching more stats, but by teaching statistical thinking in the broader sense, as well as scientific integrity and humility. In the words of Gelman (2018): “Forget about getting definitive results from a single experiment; instead embrace variation, accept uncertainty, and learn what you can.” And the best way to embrace variation and to accept uncertainty is by simulating data (McElreath, 2020; DeBruine and Barr, 2021; Vasishth and Gelman, 2021).

Simulations can be done using synthetic or real data. With data sharing and large studies on the rise, many useful questions can be addressed using data-driven simulations. It's a great time to be a data parasite! Real data have the advantage of providing rich shape information and within-participant correlations that can be tricky to simulate. So, given the need for large datasets for running simulations, and the benefits of doing so, this is another reason for scientists to release their data and code.

And we really need more datasets, particularly large multisite ones. Indeed, in the examples covered above, we only considered variance across trials and participants, but there are many more sources of variance we need to account for: for instance, variance across stimuli, study parameters, types of equipment, sites, cultures, and time of day. Without a good handle on all these sources of variability, we face a deep crisis of generalizability (Yarkoni, 2022). Maybe we should have lengthy and passionate discussions about sources of variability and how to improve the precision of our measurements, instead of wasting oxygen discussing *p* values.

## Data Availability

All the figures and analyses presented in this article can be reproduced using notebooks in the R programming language (R Core Team, 2021), as part of a reproducibility package available on GitHub at https://github.com/GRousselet/sampdist. All the figures are licensed CC-BY 4.0. Each figure caption ends with the name of the RMarkdown file that can be used to reproduce it. Some of the examples presented in this article were previously posted in a different format as blog posts covering: sampling distributions of correlations (https://garstats.wordpress.com/2018/06/01/smallncorr/) and what happens when the results are conditioned on *p* values (https://garstats.wordpress.com/2018/06/22/corrcondpval/), reaction time sampling distributions (https://garstats.wordpress.com/2018/01/24/10000/), and illustrations of estimation precision (https://garstats.wordpress.com/2018/08/27/precision/). The main R packages used to generate the data and to make the figures and notebooks are *ggplot2* (Wickham, 2016), *cowplot* (Wilke, 2017), *Cairo* (Urbanek and Horner, 2019), *dplyr* (Wickham et al., 2019), *Rfast* (Papadakis et al., 2019), *tibble* (Müller and Wickham, 2018), *rogme* (Rousselet et al., 2017), *knitr* (Xie, 2018), and the essential *beepr* (Bååth, 2018).

## Synthesis

Reviewing Editor: Catherine Schevon, Columbia University Decisions are customarily a result of the Reviewing Editor and the peer reviewers coming together and discussing their recommendations until a consensus is reached. When revisions are invited, a fact-based synthesis statement explaining their decision and outlining what is needed to prepare a revision will be listed below. The following reviewer(s) agreed to reveal their identity: Robert Calin-Jageman.

This paper provides a tutorial and a commentary, explaining how to use simulated sampling distributions to build sharper and more nuanced statistical thinking. The paper has sections discussing estimating correlations, proportions correct for performance-based outcomes, and time-based measurements (ERP latency and reaction times) - all 3 very common types of outcome measures in eNeuro papers. The paper is clear, well-written, and draws out numerous statistical insights that readers of eNeuro will likely find compelling. Although the material included in the manuscript is not novel, this is appropriate given the educational purpose of the article. All the underling code is available and well documented.

The reviewers agreed that the paper is suitable for publication, provided that the following minor modifications are made:

1. Please ensure that the purpose of the paper, and the fact that it contains no new information, is clear in the abstract.

2. Within the section on reaction times there is a passage on a one of the pitfalls of data-driven simulations, drawing on Burns et al. 2025. I found this passage difficult to follow: it comes at the end of a paper already requiring careful attention, and the setup for the question and the resulting simulation to answer the question is quite complex. Overall, I'm not sure the space available really allows the reader to absorb the lesson well - this may well be a topic that really needs its own paper or tutorial. Though it is always painful to cut a passage that has required extensive work, I ask the author to strike this section in favor of an exhortation for readers to consult Burns et al. (2025).

3. Please edit the manuscript throughout to soften the tone, in order to make it more suitable to the context of the eNeuro readership. A list of specific suggestions is included below, but please look for other similar instances requiring editing.

• Abstract - "most statistical inferences in psychology"... might want to amend to "psychology and neuroscience" to ensure readers of the abstract don't draw the wrong conclusion that the paper is directed primarily to psychologists

• Statement of significance: "that many researchers believe that nature owes them simple explanations" this seems a bit strong, and I'm also not sure if it captures the issue well (much of the rest of the paper seems to diagnose the issue as lack of knowledge/insight... this statement makes it sound more like a willful corruption of epistemic standards)

• "The typical psychology or neuroscience article reports data from one or a very few Experiments". I'm not sure if this is true in general, but it is not true for eNeuro - some papers report a single experiment, but the majority report a series of interconnected experiments. On the other hand, internal replication is rare for papers in eNeuro, so the point that researchers often leap from a single experiment to a sweeping causal claim would be accurate for this journal (and others). Just suggesting a slight rephrase here so that a reader skeptical of your position doesn't immediately jump on this claim.

• "This important point is discussed in any decent introductory frequentist statistics textbook (Howell, 2013)". - this is a bit tendentious. Perhaps something like "Sampling distributions are foundational to frequentists statistics, though often hard to grasp and so worth exploring in detail through simulations"... or something like that. The reference to Howell should probably be ".e.g. Howell, 2013)' as an example of the coverage, not as a source for the fact that sampling distributions are covered in decent stats textbooks.

• "First, they are skewed, " - just to make this clear, perhaps "First, they are skewed when drawn from a lognormal population..."

• "And that's the main reason to use large sample sizes: to get closer to the truth." .. perhaps "to get (on average) closer to the truth"?

• For "There is nothing new here: inflated effect sizes are a natural outcome of studies with small sample sizes" - perhaps "inaccurate effect sizes are a natural outcome" (with the specific push towards inflation coming by then applying a significance filter, right?)

• "Why 70%? Why {plus minus}0.1? This is not different from any thresholds used in statistics: for instance, what's the rational for p{less than or equal to}0.05, 95% confidence intervals, 80% power, Bayes factor > 3, and the inane trichotomisation of Cohen's d, other than habit?" - this is a bit of a mini-rant, and I'm not sure it helps support the point you want to make. Could just say these are possible points of reference but the full plots allow researchers to think critically about the sample-size accuracy tradeoffs inherent in their designs.

• You mentioned that the default choice of Pearson's r is poor. Perhaps the paragraph that starts with "Of course, the values I used above are completely arbitrary." Could end with something like "In addition to consulting Figure 4A, researchers may also want to explore alternatives to Pearson's r that are more robust (references)"

• I think you could consider cutting from "Not surprisingly..." up to "Indeed, consider", including Figure 4B. Specifically, I think there is so little planning currently in eNeuro papers and so little understanding of planning for power that I don't know the paragraph and figure are that useful, and you could move right on to demonstrating how adding the significance filter to these sampling distributions pollutes the literature.

• Correlations in the wild - a large, pre-registered study of brain-size and IQ found a slightly weaker correlation than even the weak one in the meta-analysis you cite (Nave et al., 2018; r = .19).. just pointing that out as another instance where the meta-analytic effect size is likely inflated. ☹

• "popular analyses, that of proportion correct data." - for this journal, you might add a sentence noting that this type of data often comes up in both human and animal experiments involving memory performance, navigation, and other performance-based outcomes

• "Let's consider" - it took me a bit of time to work through this paragraph. I suggest some slight edits for clarity that you might consider

o "imagine a participant who has a true performance level of 10% correct" (or something like that...that you are defining a true subject-level parameter is just a bit unclear as is)

o "imagine we will measure this participant's performance across 100 trials... what is the range of *actual* performance we might observe... we can find out by simulating thousands of such experiments... " - that is, I am suggesting going just a bit more slowly here, identifying the subject parameter, the number of trials, and the simulation strategy just a bit more clearly so that the reader can take it all in.

• Figure 7C and its description - I believe these are basically simulated prediction intervals. Worth a sentence to say that prediction intervals are a useful tool for setting expectations about expected variation between experiments with perhaps a reference?

• "I would argue that we have a crisis of over-confidence, one that can be tackled not by teaching more stats, but by teaching statistical thinking in the broader sense, but also scientific integrity and humility." - perhaps "as well as scientific integrity and humility"

• "within participant correlations" perhaps "within-participant correlations"

• "I leave you with an example."... I am not sure this paragraph add a lot. We have all seen presentations that we find maddening; I'm not sure this particular anecdote connects that well with the general points you've made. Also, I think the previous two paragraphs are quite good and make an excellent coda for the paper.
